# Nutritional status, dietary quality and eating disturbance issues among people with dementia in Vietnam: evidence of a cross-sectional study

**DOI:** 10.1186/s41043-024-00570-y

**Published:** 2024-07-10

**Authors:** Huong Thi Le, Anh Kim Dang, Linh Thao Thi Le, Ha Thu Thi Nguyen, Giang Thu Nguyen, Huong Thi Thu Nguyen, Hanh Bich Thi Phan, Tuan Anh Nguyen, Louise Robinson

**Affiliations:** 1 School of Preventive Medicine and Public Health, Hanoi Medical University, Hanoi, 100000 Vietnam; 2 Queensland Alliance for Environmental Health Sciences (QAEHS), The University of Queensland, 20 Cornwall Street, Woolloongabba, Brisbane, Australia; 3 Population Health Sciences Institute, Faculty of Medical Science, Newcastle University, Newcastle upon Tyne, UK; 4 Department of Geriatrics, Hanoi Medical University, Hanoi, Vietnam; 5 Scientific Research Department, National Geriatric Hospital, Hanoi, Vietnam; 6 Faculty of Dentistry, University of Medicine and Pharmacy, Vietnam National University, Hanoi, Vietnam; 7 Social Gerontology Division, National Ageing Research Institute, Melbourne, VIC Australia; 8 Department of Psychological Sciences, School of Health Sciences, Swinburne University of Technology, Melbourne, VIC Australia; 9 Quality Use of Medicines and Pharmacy Research Centre, UniSA Clinical & Health Sciences, University of South Australia, Adelaide, South Australia Australia

**Keywords:** Dementia, Nutritional status, Dietary quality, Eating disturbance

## Abstract

**Background:**

Due to cognitive impairments, people with dementia (PWD) often have difficulties in eating and drinking. This study aimed to assess the nutritional status, dietary quality and eating disturbance issues among PWD in Vietnam.

**Methods:**

We conducted a cross-sectional study at the Vietnamese National Geriatric Hospital from April to December 2022. We used Mini-Mental State Exam (MMSE) to classify the severity levels of dementia. Mini Nutritional Assessment (MNA), 24-hour recall, eating disturbance questionnaires, and anthropometric indicators were used to evaluate the nutritional status, dietary quality, and eating disorders of study subjects.

**Results:**

Overall, among 63 study participants, 74.6 per cent of PWD were at risk of or having malnutrition. By dementia classification according to MMSE scale, people with moderate and severe dementia accounted for 53.3 per cent of those who met the recommended energy levels, compared to 42.4 per cent of people with mild dementia and normal people. In the above two groups, around three per cent of participants reached the recommended amount of fibre. Calcium (50–70%), vitamin A (80–90%), and D (90%) were found to be the most severe deficiency forms of minerals and vitamins in both male and female participants. The majority of participants (90.5%) had at least one form of eating disorders with the most frequent issue being appetite changes (76.2%) and swallowing issues (50.8%).

**Conclusions:**

PWD in our sample frequently experienced malnutrition, a lack of essential nutrients, difficulties swallowing, changes in eating habits and appetite. It is neccesary to early screen and assess nutritional status and swallowing disturbance in PWD, and instruct their caregivers to prepare nutritious meals for them.

**Supplementary Information:**

The online version contains supplementary material available at 10.1186/s41043-024-00570-y.

## Introduction

Globally, dementia is one of the leading causes of dependency and impairment in the older population. Overall, the number of people with dementia (PWD) worldwide is increasing at an alarming rate, from an estimated 50 million PWD in 2020 to 82 million in 2030 and 152 million by 2050 [1]. As one of the regions experiencing the fastest growth in the older population [2], Southest Asia is forecasted to stand with significant growth in the incidence of dementia. A current systematic review of Poon et al., (2020) estimated that the number of PWD in Southeast Asia Region in 2015 was 5.51 million, with projections of 6.66 million in 2020 and 9.6 million in 2030 [3]. In Vietnam, according to the first National Dementia Conference, the number of individuals in Vietnam affected by dementia is projected to rise up to 1.2 million by 2030, from an estimation of 660,000 in 2015 [4]. Progressive dementia will result in more severe repercussions as its incidence rises. This syndrome has caused physical, psychological, social and economic impacts on PWD, their families, caregivers, and society at large [5, 6].

PWD are facing many serious nutritional problems including weight loss, malnutrition, poor dietary intake, and eating disturbances. Indeed, PWD frequently struggles to eat and drink due to physical limitations, mental and cognitive deficits, as well as psychological issues including depression and agitation [7]. According to the European Society for Clinical Nutrition and Metabolism (ESPEN), the development of dementia can cause a loss of the ability to eat efficiently, a loss of ingrained eating habits, an increase in energy requirements, and a shift in eating patterns, which can result in a smaller variety of meals and an unbalanced nutritional intake [6]. The ability to swallow, drink, and eat is typically the last function to be lost in PWD, with dire implications [6]. Finally, a vicious cycle of dementia and general malnutrition has been set off [6], which has added exacerbation of health conditions, increased mortality, and lowered the patients’ and caregivers’ quality of life.

Worldwide, eating problems in PWD have been reported in previous studies, although those studies have focused on only one or a few specific issues. Malnutrition and weight loss have been widely recognized as important clinical characteristics of dementia, with a higher frequency in older individuals [6, 8]. A systematic review and meta-analysis reported that the pooled prevalence of malnutrition and the risk of malnutrition among PWD in long-term care was 26.98 per cent and 57.43 per cent, respectively, using the Mini Nutritional Assessment (MNA) tool [8]. Eating disturbances are a natural part of advanced dementia progression [9]. A previous cohort study in Japan found that eating disturbances were observed in more than 80 per cent of people with Alzheimer’s disease (AD) [10], which is the most common type of dementia [1]. PWD frequently struggles to maintain an adequate intake of food and fluids, while there are basic self-care requirements for humans [11]. An 1-year longitudinal study reported that dietary intakes by people with AD are much less compared to cognitively intact age-matched controls, with significant differences in energy, macronutrients, calcium, iron, zinc, vitamin K, A, and dietary fiber [12].

In Vietnam, previous studies have shown that older adults with dementia often experienced malnutrition and were at risk of malnutrition [13–15]. Using the MNA, a cross-sectional study in 2021 found that among inpatient PWD at the Vietnamese National Geriatric Hospital, malnutrition was present in 66 per cent of the participants and that malnutrition risk was 29 per cent of the time [13]. Another cross-sectional study in 2023 in the same location indicated that the prevalence of malnutrition was 18.4 per cent, and the risk of malnutrition was 59.8 per cent [15]. However, to date, evidence on malnutrition and other eating difficulties in PWD in Vietnam has not well reported. Thus, it is critical to comprehensively assess the nutritional status, dietary intake, and identify eating disorder problems that PWD have faced. Therefore, this study was conducted to assess the nutritional status, dietary quality and eating disturbance issues of PWD with different severity levels in Vietnam.

## Methods

### Study participants

This study was conducted in inpatient and outpatient PWD attending the Vietnamese National Geriatric Hospital from April to December 2022. The eligible criteria for recruiting participants included (a) having a formal diagnosis with dementia using Diagnostic and Statistical Manual of Mental Disorders, Fifth Edition (DSM-V) criteria (2013) [16] by specialists; (b) having full medical records; (c) providing informed consent to participate in the study. Exclusion criteria included: (a) having medical conditions that can affect the nutritional status of people with dementia such as fever, cancer, acute illness, severe food allergy, severe digestive disorder; or (b) having major psychiatric disorders or neurological disorders or developmental abnormalities other than dementia, which may influence their smell and taste; or (c) patients requiring special therapeutic diets.

In this study, we used the sample size formula for estimating a proportion. By applying *p* = 0.95 (the percentage of PWD having malnutrition using MNA [13]), ε = 0.06 and α = 0.05, the sample size was 56. We also added a refusal rate of 10 per cent and the final number was 61 subjects. In fact, the total of 63 PWD took part in this study. All PWD attending the hospital during the study period, who met the inclusion criteria were chosen for the study until the sample size was reached.

### Measurement and instruments

The trained investigators collected the data using an interview-administered questionnaire. It took about 20 min to complete the whole questionnaire, and all results were recorded and managed using REDCap software. The Eating Disturbance Issues questionnaire was translated into Vietnamese by one researcher (LTTL) then translated back to English by two senior researchers (AKD and GTN). After this step, we conducted a pilot study among nine PWD who had various demographic characteristics to test the questionnaire. Based on the basis of participants’ comments, small phrasing adjustments were made. The official questionnaire in this study included six main parts:

#### Demographic characteristics

PWD age, gender, current occupation, educational attainment, people whom PWD is living with (family/caregivers/live alone/others), living area.

#### The mini mental state exam (MMSE)

Mini Mental State Exam (MMSE) has widely used to evaluate the severity of dementia in clinical and research settings in the world [17]. It has demonstrated significant efficacy in detecting dementia among elderly people in the Vietnamese community [18]. This examination had 11 items covering memory, calculation, attention, recollection and assessing the ability to name, follow verbal and written orders, compose a statement on the spot, and duplicate a complex polygon comparable to a Bender-Gestalt Figure. The test result was classified as following: (a) 0–9 points as severe dementia, (b) 10–17 points as moderate dementia, (c) 18–23 points as mild dementia, and (d) ≥ 24 points as normal. A score of 0 was the lowest and 30 was the highest possible. The higher the score, the better the participant’s cognitive performance [17].

#### Anthropometric indices

We measured anthropometric indicators such as weight, height, BMI, mid-upper arm circumference (MUAC), muscle mass, body fat percentage. Specific techniques were described as follows.

- Weight of patients was measured using the Tanita scale (BC 758), with the accuracy of 0.1 kg. Participants stood in the middle of the scale; their eyes looked straightly, weight was evenly distributed on both legs. The scale was placed in a stable and flat position.
- Vertical height of patients was measured with a wooden ruler with the accuracy of 1 mm. Participants took off shoes, and their heels, legs, buttocks, shoulders, heads stayed in a straight line against the vertical ruler, while their eyes looked straight in a horizontal line. Two hands hung along his/her sides.
- BMI was calculated by dividing weight in kilograms to height in meters squared, in the metric system. Using WHO classification (1998) for adults, BMI < 18.5 is considered as underweight, 18.5 ≤ BMI ≤ 24.9 as normal range, 25 ≤ BMI ≤ 29.9 as overweight, BMI ≥ 30 as obesity [19].
- To measure the MUAC, we used a soft, non-stretch ruler with an accuracy of 1 mm. We determined the point which was the middle of the apex of the shoulder blade and the apex of the brachial ridge of the left mid-arm.
- To determine muscle mass (kg) and body fat percentage (BF%) (%), we used bioelectrical impedance analysis (BIA) of Tanita scale (BC 758). For the scale to display the results, the patient must stand upright on the scale for at least 10–20 s.

#### Mini nutritional assessment (MNA)

MNA-LF (Mini Nutritional Assessment – Long Form) was applied to screen and assess the nutritional status of people 65 years and older. The MNA was developed and validated with the purpose of offering geriatric patients in clinics, hospitals, and nursing homes a solitary, expeditious evaluation of their nutritional status [20]. Many previous studies in Vietnam applied MNA as a global tool to assess malnutrition risk in older adults in multiple settings [14, 20, 21]. The questionnaire had two sections, including screening and assessment parts. The first part consists of six items about weight loss, food intake, neuropsychological problems, psychological stress or acute disease, mobility and Body Mass Index (BMI). The second part focuse*s* on various aspects such as independent living, medication usage, ulcers, diet, food and fluid consumption, mode of feeding, self health report, mid-arm and calf cicumference. The total MNA score of both parts is 30. Nutritional status of PWD was categorized as followed: under 17 points as malnutrition, 17-23.5 as at risk of malnutrition, and 24–30 as normal nutritional status [20].

To measure calf circumference (CC) in the MNA scale, we used a soft, non-stretch measurement tape with an accuracy of 1 mm. The measurement was performed at the widest part of the calf, in the sitting position with their bare feet down, with participants’ legs bent to 90 degrees.

#### 24-hour food intake

Participants were asked to report their dietary intake within past 24 h in three typical days (not holidays, anniversaries, parties, birthdays, fasting days, or any special-diet days) include all meals, snacks, and beverages consumed. The balance of the diet was also assessed including daily energy intake in kcal (EN%), as well as protein, fibre, fat intake, and micronutrients.

#### Eating disturbance issues questionnaire

Eating disturbance issues questionnaire, which is newly used to assess characteristics of eating disturbance in PWD, had been originally designed by Ikeda et al. (2002) [23] and had been adapted by Kai et al. (2015) [10]. It contains 37 questions covering 5 issues/behaviors namely (a) swallowing disturbance, (b) appetite changes, (c) food preference including sweet food preference and food fads, (d) eating habits including stereotypic eating behaviors and decline in table manners, and (e) other eating behaviors including food cramming and indiscriminate eating. To determine the presence or absence of a certain issue, we determined the difference between premorbid condition and the most recent month’s assessment. If the patient had significant change in any symptom, we would determine that symptom is present [10].

### Data analysis

The data analysis was conducted using Stata 16.0 software. Descriptive statistics were performed through the calculation of mean values, standard deviations (SD) for quantitative variables and frequency, percentage for nominal/categorical variables and ordinal variables) Inference statistics were used to compare the differences in nutritional status and eating disturbance issues among patients with different severity levels of dementia according to MMSE. A *p*-value ≤ 0.05 was considered statistically significant for all analyzes. In addition, we expressed the effect size (ES), which refers to the degree of the disparity observed between groups [24], by different coefficients corresponding to different tests.

In analyzing nutritional status and dietary quality, it is necessary to comprehend the differences between two severity levels of dementia as mild (early stage) and severe (advanced stage) for appropriate nutritional consultation and development of nutritional intervention programs [6]. For nutritional status, we used independent t-test and Mann-Whitney test to examine the differences in nutritional status and anthropometric indices across two dementia groups based on MMSE scores: moderate/severe (MMSE ≤ 17 points) and normal/mild (MMSE ≥ 18 points).

Regarding to 24-hour food intake, we described the number and proportion of people meeting the recommended needs according to two genders (male and female) and according to two dementia groups (normal - mild, and moderate – severe). In addition, daily intakes of food items and nutrients were calculated using the Vietnamese food composition Table (2019) [25]. The amount of nutrient was compared to the recommendation according to ESPEN guideline on clinical nutrition and hydration in geriatrics [26] and Dietary Guidelines for Americans 2020–2025, 9th version [27].

In terms of eating disturbance, a symptom was considered positive if it was rated as frequent (about once per week) to very frequent (one or more times per day or continuously). Any symptoms of any domain including swallowing disturbance, appetite changes, food preference, eating habits, and eating behaviors were reported, that domain was marked as positive. In addition, because “loss of appetite” and “increase in appetite” may have distinct neurological origins, these two symptoms were studied independently in the domain of “change of appetite” [10]. We used χ2 test and Fisher’s exact test to evaluate the differences in each domain of eating disorder issues between four dementia groups including normal, mild, moderate, and severe, similar to the classification of dementia groups in previous study [10].

### Ethical consideration

Prior to the data collection, written informed consent was provided to PWD or their families/main caregivers. Participants can refuse or withdraw from the study at any time, without any impact on their treatment progress. All information on research subjects was kept confidential for research purposes only. The study was approved by the Institutional Ethical Review Board of Hanoi Medical University (code 509/GCN-HDDDNCYSH-DHYHN). This study was supported by the National Institutes of Health (NIH) R01AG064688 grant (Hinton/Nguyen MPI). The content of this study is solely the responsibility of the authors and does not necessarily represent the official views of the NIH.

## Results

Among 63 participants, females accounted for 68.2 per cent, and about three-fourths of participants (74.6%) lived in urban areas. At the time of studying, the percentage of people with mild, moderate and severe dementia were 33.33%, 31.8% and 15.9%, respectively. The mean age was 74.7 ± 7.3 (years), and the average MMSE score was 17.1 ± 7.0 *(Appendix S1).*

**Table 1:** Nutritional status of study subjects by the severity levels of dementia (*n* = 63)

Nutritional status	Total (*n* = 63)		Moderate and Severe (*n* = 30)		Normal and Mild (*n* = 33)		*p-value*	ES
	*n*	%	*n*	%	*n*	%		
**Nutritional status according to MNA**								
***Malnutrition***	10	15.9	6	20.0	4	12.1	0.53^C^	0.1429^W^
***At risk of malnutrition***	37	58.7	18	60.0	19	57.6		
***Normal***	16	25.4	6	20.0	10	30.3		
**Nutritional status according to BMI**								
***Underweight***	2	3.2	1	3.3	1	3.0	0.15^F^	0.2578^V^
***Normal***	38	60.3	15	50.0	23	69.7		
***Overweight***	22	34.9	14	46.7	8	24.2		
***Obese***	1	1.6	0	0	1	3.0		
Nutritional indicators	*Mean*	*SD*	*Mean*	*SD*	*Mean*	*SD*	*p-value*	ES
**MNA score**	20.5	3.7	19.8	3.6	21.1	3.8	0.11^T^	0.35 (-0.15–0.85)^D^
**Weight (kg)**	55.5	8.9	56.6	9.7	54.6	8.2	0.72	-0.22 (-0.72 -0.28)^D^
**Height (m)**	1.5	0.1	1.5	0.1	1.5	0.1	0.23^M^	-0.04^R^
**BMI (kg/m2)**	24.4	5.5	23.9	3.2	24.8	7.1	0.72^M^	-0.01^R^
**MUAC (cm)**	27.3	3.4	27.4	3.0	27.3	3.7	0.97	-0.01 (-0.50–0.49)^D^
**Muscle mass (kg)**	35.0	7.3	36.5	8.3	33.8	6.2	0.30^M^	-0.03^R^
**Body fat percentage (%)**	32.4	7.3	31.6	7.5	33.1	7.2	0.45	0.20 (-0.31–0.70)^D^

*Notes: MNA: Mini Nutritional Assessment; BMI: Body Mass Index; MUAC: mid-upper arm circumference; ES: effect size;*^*C*^: *Chi-square test;*^*F*^: *Fisher’s exact test;*^*T*^: *t-test;*^*M*^: *Mann-Whitney test;*^*W*^: *Cohen’s W;*^*V*^: *Cramer’s V;*^*D*^: *Cohen’s D (95%CI);*^*R*^: *Rank-biserial correlation coefficient*

Table 1 illustrates the nutritional status of study subjects by severity levels of dementia. According to MNA scale, 74.6% participants suffered from malnutrition or were at risk of malnutrition. Using BMI, the number of underweight was 3.2%. The mean BMI and MNA score of subjects were 24.4 ± 5.5 and 20.5 ± 3.7, respectively. Other anthropometric variables include weight (55.5 ± 8.9, kg), height (1.5 ± 0.1, m), MUAC (27.3 ± 3.4, cm), muscle mass (35.0 ± 7.3, kg), body fat percentage (32.4 ± 7.3, %). There were no statistically significant differences in nutritional indicators between two groups of normal/ mild dementia and moderate/severe dementia.

**Table 2:** The percentage of people with dementia meeting energy, protein, and fibre recommendations by dementia levels (*n* = 63)

Intake	Dementia levels according to MMSE															
	*Moderate and severe dementia (n = 30)*								*Mild dementia and normal (n = 33)*							
	Mean	SD	% of meeting recommendation*						Mean	SD	% of meeting recommendation*					
			**< 50%**		**50–75%**		**> 75%**				**< 50%**		**50–75%**		**> 75%**	
			***n***	***%***	***n***	***%***	***n***	***%***			***n***	***%***	***n***	***%***	***n***	***%***
***Energy***	1351.3	557.4	5	16.7	9	30.0	16	53.3	1207.2	561.6	10	30.3	9	27.3	14	42.4
***Protein***	60.9	27.2	2	6.7	7	23.3	21	70.0	62.2	30.3	1	3.0	7	21.2	25	75.8
***Fibre***	6.3	5.5	27	90.0	2	6.7	1	3.3	6.1	4.5	30	90.9	2	6.1	1	3.0

Notes: *Recommendation according to ESPEN guidelines on clinical nutrition and hydration in geriatrics by Volkert et al. (2019), with 30 kcal/kg/day for the total energy in a day, 1 g protein/kg/day, and 25 g fibre/day. MMSE: Mini-Mental State Exam

Table 2 presents the percentage of PWD meeting nutritional dietary recommendations. Among people with moderate and severe dementia, more than half of the participants met the energy recommendations, which was higher than that of mild dementia and normal group (42.4%). In addition, a large percentage of participants in both groups reached the recommended protein (70.0% and 75.8%, respectively). By contrast, a low proportion of all study subjects reached the recommended fibre intake (3.3% and 3.0%, respectively).

Regarding to minerals and vitamins, nutrients deficiencies in the diet of PWD varied depending on gender and severity of the disease. Among women with moderate and severe dementia, most subjects did not meet calcium, folate, vitamin A, D recommendations (50%, 55%, 80%, 90%, respectively), while among women with mild dementia or normal cognition, most participants did not meet the required needs of calcium (60.9%), magnesium and folate (56.5%), vitamin A (82.6%), and D (87%) *(Appendix S2).* Meanwhile, among men with moderate and severe dementia, 50% met recommended calcium, 70% met magnesium, 80% met vitamin A, and 90% met vitamin D level. Among men with mild dementia or normal cognition, the most deficient nutrients in their diets were vitamin B2 and PP (50%), potassium, zinc and folate (60%), calcium and vitamin B12 (70%), magnesium, vitamin A and D (90%) *(Appendix S3).*

Table 3 describes the frequency and percentage of PWD having eating disturbance issues and its components by severity levels of dementia. Among 63 individuals, 90.5% of people had at least one eating disturbance issue. In five domains, “appetite changes” accounted for the largest proportion (76.2%), followed by “eating habits” (60.3%), “Swallowing disturbance” and “food preferences” (50.8%). However, there were no statistically significant differences in the prevalence of eating problems between four levels of dementia including normal, mild, moderate and severe.

**Table 3:** The frequency and percentage of study subjects having eating disturbance issues by severity levels of dementia (*n* = 63)

Categories	Normal (*n* = 12)	Mild (*n* = 21)	Moderate (*n* = 20)	Severe (*n* = 10)	Total (*n* = 63)	*p*-value	ES
	*n* (%)	*n* (%)	*n* (%)	*n* (%)	*n* (%)		
**Eating disturbance issues**	9 (75.0)	19 (90.5)	20 (100)	9 (90.0)	57 (90.5)	0.10^F^	0.29^V^
**Swallowing disturbance**	6 (50.0)	9 (42.9)	11 (55.0)	6 (60.0)	32 (50.8)	0.80^C^	0.13^W^
**Appetite change**	9 (75.0)	15 (71.4)	17 (85.0)	7 (70.0)	48 (76.2)	0.71^F^	0.15^V^
**Loss of appetite**	2 (16.7)	9 (42.9)	9 (45.0)	2 (20.0)	22 (34.9)	0.26^F^	0.26^V^
**Increase in appetite**	6 (50.0)	4 (19.0)	4 (20.0)	1 (10.0)	15 (23.8)	0.15^F^	0.31^V^
**Food preference**	4 (33.3)	11 (52.4)	14 (70.0)	3 (30.0)	32 (50.8)	0.10^C^	0.31^W^
**Eating habits**	5 (41.7)	11 (52.4)	15 (75.0)	7 (70.0)	38 (60.3)	0.23^F^	0.27^V^
**Other eating behaviors**	3 (25.0)	8 (38.1)	7 (35.0)	6 (60.0)	24 (38.1)	0.43^F^	0.22^V^

Note: ^*F*^: *Fisher’s exact test;*^*C*^: *Chi-square test; ES: Effect size; V: effect sizes are expressed as Cramer’s V; W: effect sizes are expressed as Cohen’s W*

**Fig. 1:**
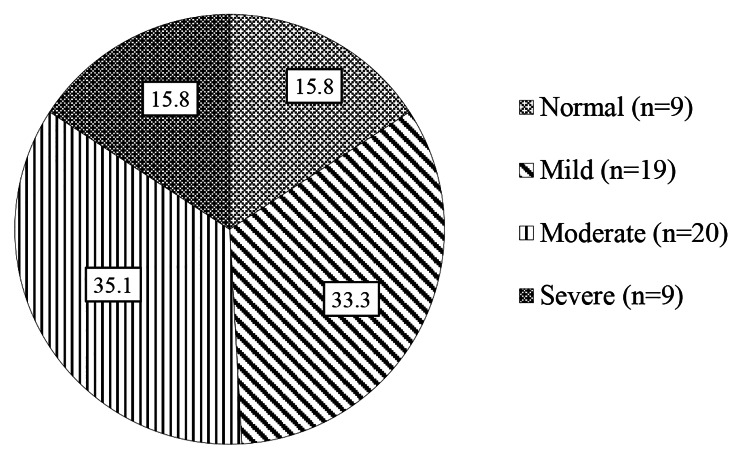
The proportion of people with dementia having eating disturbance issues (*n* = 57)

Figure 1 shows the proportion of PWD having eating disturbance issues. The group with the most eating disorders was those with moderate dementia (35.1%).

## Discussion

To the best of our knowledge, our study is one of the first studies in Vietnam evaluating the nutritional status, balance of dietary intake and several eating problems of PWD. The findings provide valuable evidence to clinicians, public health experts, and policy makers in early screening nutritional status, identifying main eating difficulties and proposing dietary recommendations for PWD in Vietnam. This research showed a significant frequency of PWD being malnourished or at risk of malnutrition. Regardless of gender or dementia severity, the diets of PWD in Vietnam were particularly low in calcium, vitamins A and D. PWD faced a lot of trouble swallowing and their tastes shift, among other eating and drinking challenges.

We found a relatively high percentage of people being malnourished or at risk of malnutrition, according to MNA. Our figure was higher than that of earlier cluster randomized clinical trials [28, 29] (37% and 47.8%, respectively). However, our figure was lower than not only that of some previous studies in other countries such as Finland (91%) [30], Spain (97.6%) [31], but also that of a prior study in the same location as ours [13] (95%). Our finding is similar to that of a cross-sectional study in Belgium [32], which reported that 76% of PWD being malnourished of at risk of malnourishment. In general, we concur with the prior statement that malnutrition is a concern for PWD [6]. PWD frequently have behavioural and psychological symptoms, making them consume fewer food or liquids. In addition, PWD in their final stages depend on others to assist them to eat and drink [33]. Their dietary intake may be negatively impacted by this reliance, which could also worsen malnutrition and its symptoms [34]. Given the strong connection between malnutrition and morbidity as well as mortality in older adults [35], it is crucial to minimize the risk of malnutrition in PWD, especially older adults, as soon as possible.

In both two groups of normal/mild dementia and moderate/severe dementia, most of PWD consumed sufficient recommended amounts of protein, but the percentage of people who meet their calorie and fibre needs was at an average and very low level, respectively. Insufficient energy may be linked to sarcopenia and frailty [36], which contributed to the weight loss, muscle wastage and is linked to a higher risk of institutionalization, morbidity, mortality [37, 38], falls, impairments, and fractures [39]. In addition, to the extent that all national dietary recommendations and the food pyramid for the older adults emphasize the need to increase dietary fibre intake and, by extension, fruits and vegetables, it appears that fibre intake is particularly important for the older adults, because it acts as prebiotics on microbiota health in the intestinal tract [40].

PWD in Vietnam typically consume low-micronutrient diets. Among minerals and vitamins in the diets of our study subjects, calcium, vitamin A, and D were more often deficient, regardless of gender and severity levels of dementia. The repercussions of not getting enough of these nutrients in the diet can be very severe, especially for older people. Firstly, regarding to calcium and vitamin D, persons 65 years and older are at a significant risk of developing fat-soluble vitamin D because of the decline in sun exposure [41]. This can lead to osteoporosis and those who have both dementia and osteoporosis have worsen health outcomes [42]. The risk of osteoporosis appears to be reduced by consuming enough vitamin D and calcium throughout a person’s life [43]. Our findings contributed to the body of knowledge indicating that the older people with dementia in Vietnam have a higher risk of fracture and osteoporosis. Secondly, in terms of vitamin A, it plays various crucial functions in oxidation and immunological function, which are the two fundamental aspects of ageing in males as well as females [44]. Since vitamin A may be easily supplied from food as well as dietary supplements, vitamin A insufficiency in older people is uncommon in the United States (US) [45]. However, our research demonstrates the opposite; one possible reason may be Vietnamese people’s tendency of eating less and paying less attention to foods high in β-carotene and vitamin A, as well as using dietary supplements less frequently.

The percentage of PWD having at least one eating disturbance issues was high, which was higher than that in a prospective hospital-based cohort study in Japan [10]. This difference might be because our research participants were PWD (including AD and other types of dementia), whereas the subjects in the prior study were only people with AD. AD is the most common cause of dementia [1], while there are many other forms of dementia that affect eating and drinking problems [23]. In addition, eating disorders are a known natural component of dementia development [9]. During the course of disease, patients show motor agitation, behavioural abnormalities, and cognitive deterioration. As symptoms intensify, this frequently causes issues with eating and drinking [46]. Although most of our study participants fell into the group of mild dementia, the high prevalence of eating problems may indicate an increased possibility of dementia among PWD in our sample in the future. However, further studies are still needed to accurately predict the risk of advanced dementia in these people.

Among eating issues, our study found that swallowing disturbance, eating habits and appetite changes were the most common eating problems among PWD. These findings were similar to those of previous studies, indicating the high prevalence of PWD having difficulty swallowing or dysphagia [10, 47, 48], which caused by the lessened tongue movement and delayed swallowing response [49, 50]. Since difficulties swallowing directly affect how much food is consumed, it may result in a progressive decrease in the amount of solid and liquid food, weight loss, malnutrition, dehydration, more prolonged hospital stays, and higher medical expenses [48, 51–53]. In addition, our results revealed certain alterations in eating that were similar to those observed in earlier studies [10, 41, 54, 55]. These changes included increased and decreased food intake, different food preferences, the use of inedible items, and interruptions non the eating process such as the sense of smell, movement, and urination, or personal hygiene [54]. These were not only observed in non-institutionalized PWD [55] but also in both inpatients and outpatients PWD, according to our study. Age-related changes in taste and smell may affect dietary preferences and restrict what types and amount of food older people consume [10, 41, 54]. Finally, PWD develop a vicious circle of health issues that includes dementia progression, eating disorders, and malnutrition. All of these factors could explain why PWD in Vietnam are more likely to experience malnutrition in our study.

Regarding to the strengths, this study is among the first to assess the nutritional status, dietary quality and eating disturbance issues among PWD in Vietnam. Our findings have the potential to contribute to future intervention studies to improve nutritional status and eating difficulties in PWD. However, several limitations should be noticed. Thelimitation in sample size may lead to the ability of comparing differences among two groups. Thus, nutritional intervention studies could utilize in a large sample size to increase the reliability of the findings. Furthermore, future studies should be conducted in different regions or different settings in Vietnam to generalise the outcome. In addition, we highly recommend that future studies should apply diverse methods to assess the dietary quality of patients such as 24-hour recording multiple times (3–7 consecutive days, investigating the frequency of consumption of food, food weighing) in order to increase the accuracy when assessing the quality of dietary intake. Finally, while the Eating Disturbance Issues questionnaire has been utilized globally, it remains novel in Vietnam. Further study is required to evaluate the reliability and validity of Vietnamese version of this tool and to implement it extensively for evaluating eating disorders in individuals with dementia.

Based on the findings, it is important to early screen and assess nutritional status of PWD, regularly check to identify swallowing issue. We recommend that health workers should provide specific explanation to caregivers about skills in nutritional care for PWD, such as food selection and preparation to make adequately nutritious meals. For PWD, eating disturbances and other challenges are unavoidable; therefore, it is essential to recognise and assist them in solving these difficulties, especially being willing to accommodate some of their dietary preferences or using appropriate eating aids for the older population.

## Conclusion

In conclusion, malnutrition, deficiencies in calcium, vitamin A and D, and swallowing disturbance are prevalent problems among people with dementia in Vietnam. It is necessary to early screen malnutrition and micro-nutrients deficiency for PWD, and personalize the nutritional intervention, such as food preferences and eating habits. Further research could be directed towards intervention strategies, including dietary preparation and feeding techniques, in an effort to enhance the nutritional status of people with dementia.

### Electronic supplementary material

Below is the link to the electronic supplementary material.

Supplementary Material 1

## Data Availability

The datasets used and/or analysed during the current study are available from the corresponding author on reasonable request.

## Abbreviations

AD: Alzheimer’s disease
BMI: Body Mass Index
ESPEN: European Society for Clinical Nutrition and Metabolism
MMSE: Mini-Mental State Exam
MNA: Mini Nutritional Assessment
MUAC: mid-upper arm circumference
PWD: people with dementia
WHO: World Health Organization
