# Supplementary material for: Nutritional status, dietary quality and eating disturbance issues among people with dementia in Vietnam: evidence of a cross-sectional study
Source: J Health Popul Nutr. 2024 Jul 10;43:107. doi: 10.1186/s41043-024-00570-y (PMC11238499; doi:10.1186/s41043-024-00570-y)
Supplement: Supplementary file 1 — Supplementary Material 1 [file 41043_2024_570_MOESM1_ESM.docx]

**SUPPLEMENTARY MATTERIALS**

**Appendix S1. Demographic characteristics of study subjects (n=63)**

| **Characteristics** | | **Frequency (n)** | **Percent (%)** |
| --- | --- | --- | --- |
| Age (Mean ± SD) | | 74.7 ± 7.3 | |
| Gender | Male | 20 | 31.8 |
|  | Female | 43 | 68.2 |
| Current occupation | Retirement | 62 | 98.4 |
|  | Working | 1 | 1.6 |
| Currently living with | Family (wife/husband/child) | 59 | 93.6 |
|  | Caregiver | 1 | 1.6 |
|  | Live alone | 2 | 3.2 |
|  | Other | 1 | 1.6 |
| Living area | Rural | 16 | 25.4 |
|  | Urban | 47 | 74.6 |
| Educational attainment | Under high school | 26 | 41.3 |
|  | High school | 12 | 19.0 |
|  | Above high school | 19 | 30.2 |
|  | Unknown | 6 | 9.5 |
| The severity of dementia according to MMSE | Normal | 12 | 19.0 |
|  | Mild dementia | 21 | 33.3 |
|  | Moderate dementia | 20 | 31.8 |
|  | Severe dementia | 10 | 15.9 |
| The average MMSE score (Mean ± SD) | | 17.1±7.0 | |

*Notes: MMSE: Mini-Mental State Exam*

**Appendix S2. The percentage of meeting minerals and vitamins recommendations in female people with dementia by severity levels of dementia according to MMSE (n=43)**

| **Intake** | **Moderate and severe dementia (n=20)** | | | | | | | | **Mild dementia and normal (n=23)** | | | | | | | |
| --- | --- | --- | --- | --- | --- | --- | --- | --- | --- | --- | --- | --- | --- | --- | --- | --- |
|  | **Mean** | **SD** | **% of meeting recommendation*** | | | | | | **Mean** | **SD** | **% of meeting recommendation*** | | | | | |
|  |  |  | **<50%** | | **50-75%** | | **>75%** | |  |  | **<50%** | | **50-75%** | | **>75%** | |
|  |  |  | ***n*** | ***%*** | ***n*** | ***%*** | ***n*** | ***%*** |  |  | ***n*** | ***%*** | ***n*** | ***%*** | ***n*** | ***%*** |
| ***Minerals*** | | | | | | | | | | | | | | | | |
| Calcium (mg/day) | 736.7 | 658.9 | 10 | 50 | 3 | 15 | 7 | 35 | 646.2 | 567.2 | 14 | 60.9 | 2 | 8.7 | 7 | 30.4 |
| Sodium (mg/day) | 556.8 | 453.1 | 18 | 90 | 2 | 10 | 0 | 0 | 589.8 | 796.5 | 20 | 87.0 | 1 | 4.3 | 2 | 8.7 |
| Potassium (mg/day) | 2041.4 | 1252.5 | 8 | 40 | 3 | 15 | 9 | 45 | 2063.2 | 1253.9 | 7 | 30.4 | 6 | 26.1 | 10 | 43.5 |
| Magnesium (mg/day) | 178.3 | 110.5 | 9 | 45 | 7 | 35 | 4 | 20 | 185.5 | 135.7 | 13 | 56.5 | 4 | 17.4 | 6 | 26.1 |
| Zinc (mg/day) | 7.3 | 4.0 | 3 | 15 | 7 | 35 | 10 | 50 | 7.7 | 3.9 | 4 | 17.4 | 4 | 17.4 | 15 | 65.2 |
| Phosphorus (mg/day) | 941.3 | 705.3 | 2 | 10 | 5 | 25 | 13 | 65 | 948.2 | 664.5 | 3 | 13.0 | 4 | 17.4 | 16 | 69.6 |
| Iron (mg/day) | 8.5 | 5.1 | 4 | 20 | 6 | 30 | 10 | 50 | 8.9 | 3.9 | 2 | 8.7 | 4 | 17.4 | 17 | 73.9 |
| ***Vitamins*** | | | | | | | | | | | | | | | | |
| Vitamin C (mg/day) | 122.8 | 111.8 | 5 | 25 | 1 | 5 | 14 | 70 | 133.5 | 115.2 | 3 | 13.0 | 5 | 21.7 | 15 | 65.2 |
| Vitamin B1 (mg/day) | 0.9 | 0.4 | 4 | 20 | 7 | 35 | 9 | 45 | 1.2 | 0.6 | 3 | 13.0 | 3 | 13.0 | 17 | 73.9 |
| Vitamin B2 (mg/day) | 1.2 | 1.1 | 6 | 30 | 4 | 20 | 10 | 50 | 1.0 | 0.9 | 11 | 47.8 | 3 | 13.0 | 9 | 39.1 |
| Vitamin PP (mg/day) | 8.8 | 4.7 | 9 | 45 | 5 | 25 | 6 | 30 | 10.4 | 4.9 | 5 | 21.7 | 8 | 34.8 | 10 | 43.5 |
| Folate (µg DFE/day) | 208.6 | 207.0 | 11 | 55 | 5 | 25 | 4 | 20 | 218.1 | 183.7 | 13 | 56.5 | 3 | 13.0 | 7 | 30.4 |
| Vitamin B12 (µg/day) | 3.1 | 4.3 | 6 | 30 | 5 | 25 | 9 | 45 | 2.2 | 2.4 | 10 | 43.5 | 5 | 21.7 | 8 | 34.8 |
| Vitamin A (µg/day) | 250.5 | 316.7 | 16 | 80 | 2 | 10 | 2 | 10 | 166.4 | 216.8 | 19 | 82.6 | 2 | 8.7 | 2 | 8.7 |
| Vitamin D (µg/day) | 4.1 | 7.0 | 18 | 90 | 1 | 5 | 1 | 5 | 3.3 | 5.8 | 20 | 87.0 | 1 | 4.3 | 2 | 8.7 |

Notes: **Recommendation according to Dietary Guidelines for Americans 2020-2025, 9th version. MMSE: Mini Mental State Exam. DFE = Dietary Folate Equivalent. AT = alpha-tocopherol*

**Appendix S3. The percentage of meeting minerals and vitamins recommendations in male people with dementia by severity levels of dementia according to MMSE (n=20)**

| **Intake** | **Moderate and severe dementia (n=10)** | | | | | | | | **Mild dementia and normal (n=10)** | | | | | | | |
| --- | --- | --- | --- | --- | --- | --- | --- | --- | --- | --- | --- | --- | --- | --- | --- | --- |
|  | **Mean** | **SD** | **% of meeting recommendation*** | | | | | | **Mean** | **SD** | **% of meeting recommendation*** | | | | | |
|  |  |  | **<50%** | | **50-75%** | | **>75%** | |  |  | **<50%** | | **50-75%** | | **>75%** | |
|  |  |  | **n** | **%** | **n** | **%** | **n** | **%** |  |  | **n** | **%** | **n** | **%** | **n** | **%** |
| ***Minerals*** | | | | | | | | | | | | | | | | |
| Calcium (mg/day) | 798.7 | 855.4 | 6 | 60 | 1 | 10 | 3 | 30 | 800.9 | 903.5 | 7 | 70 | 1 | 10 | 2 | 20 |
| Sodium (mg/day) | 518.5 | 436.1 | 9 | 90 | 1 | 10 | 0 | 0 | 448.0 | 364.2 | 10 | 100 | 0 | 0 | 0 | 0 |
| Potassium (mg/day) | 2361.3 | 789.8 | 2 | 20 | 3 | 30 | 5 | 50 | 1728.6 | 879.3 | 6 | 60 | 3 | 30 | 1 | 10 |
| Magnesium (mg/day) | 188.3 | 59.5 | 7 | 70 | 2 | 20 | 1 | 10 | 136.0 | 70.5 | 9 | 90 | 1 | 10 | 0 | 0 |
| Zinc (mg/day) | 9.8 | 2.9 | 0 | 0 | 1 | 10 | 9 | 90 | 6.2 | 3.3 | 6 | 60 | 3 | 30 | 1 | 10 |
| Phosphorus (mg/day) | 1014.8 | 369.6 | 0 | 0 | 0 | 0 | 10 | 100 | 803.8 | 650.4 | 0 | 0 | 4 | 40 | 6 | 60 |
| Iron (mg/day) | 11.2 | 2.4 | 0 | 0 | 0 | 0 | 10 | 100 | 9.8 | 3.2 | 1 | 10 | 0 | 0 | 9 | 90 |
| ***Vitamins*** | | | | | | | | | | | | | | | | |
| Vitamin C (mg/day) | 150.5 | 181.7 | 2 | 20 | 3 | 30 | 5 | 50 | 122.4 | 53.1 | 0 | 0 | 1 | 10 | 9 | 90 |
| Vitamin B1 (mg/day) | 1.4 | 0.3 | 0 | 0 | 0 | 0 | 10 | 100 | 1.0 | 0.3 | 1 | 10 | 3 | 30 | 6 | 60 |
| Vitamin B2 (mg/day) | 1.3 | 1.1 | 2 | 20 | 4 | 40 | 4 | 40 | 0.9 | 0.9 | 5 | 50 | 4 | 40 | 1 | 10 |
| Vitamin PP (mg/day) | 10.8 | 3.0 | 1 | 10 | 6 | 60 | 3 | 30 | 10.6 | 7.4 | 5 | 50 | 3 | 30 | 2 | 20 |
| Folate (µg DFE/day) | 287.2 | 120.6 | 3 | 30 | 3 | 30 | 4 | 40 | 210.1 | 218.9 | 6 | 60 | 2 | 20 | 2 | 20 |
| Vitamin B12 (µg/day) | 2.8 | 3.3 | 4 | 40 | 2 | 20 | 4 | 40 | 1.3 | 2.2 | 7 | 70 | 2 | 20 | 1 | 10 |
| Vitamin A (µg/day) | 263.4 | 191.5 | 8 | 80 | 2 | 20 | 0 | 0 | 136.9 | 295.6 | 9 | 90 | 0 | 0 | 1 | 10 |
| Vitamin D (µg/day) | 3.5 | 4.8 | 9 | 90 | 1 | 10 | 0 | 0 | 2.0 | 4.8 | 9 | 90 | 0 | 0 | 1 | 10 |

Notes: ********Recommendation according to Dietary Guidelines for Americans 2020-2025, 9th version. MMSE: Mini Mental State Exam. DFE = Dietary Folate Equivalent. AT = alpha-tocopherol*
